# Patients’ quality of life during active cancer treatment: a qualitative study

**DOI:** 10.1186/s12885-018-4868-6

**Published:** 2018-10-04

**Authors:** Jordan Sibeoni, Camille Picard, Massimiliano Orri, Mathilde Labey, Guilhem Bousquet, Laurence Verneuil, Anne Revah-Levy

**Affiliations:** 1 Service Universitaire de Psychiatrie de l’Adolescent, Argenteuil Hospital Centre, 69 rue du LTC Prud’hon, 95107 Argenteuil, France; 2 ECSTRRA Team, UMR-1153, Inserm, Paris Diderot University, Sorbonne Paris Cite, Paris, France; 3 Department of Dermatology, Caen University Hospital, F-14033 Caen, France; 4 Université de Caen Normandie, Medical School, F-14000 Caen, France; 5 McGill Group for Suicide Studies, Douglas Mental Health University Institute, Department of Psychiatry, McGill University, Montreal, Canada; 6 EPSM Lille métropole, pôle de psychiatrie adulte 59g21, Lille, France; 7 Université Paris 13 – Léonard de Vinci, Villetaneuse, France; 8 AP-HP-Hôpital Avicenne, Service d’Oncologie médicale–Bobigny, Bobigny, France

**Keywords:** Qualitative research, Oncology, Care, Physician-patient relationship, Quality of life

## Abstract

**Background:**

Patients’ quality of life has become a major objective of care in oncology. At the same time, it has become the object of increasing interest by researchers, working with both quantitative and qualitative methods. Progress in oncology has enabled more patients to survive longer, so that cancer is increasingly often a chronic disease that requires long-term treatment that can have negative effects on patients’ quality of daily life. Nonetheless, no qualitative study has explored what patients report affects their quality of daily life during the treatment period. This study is intended to fill this gap.

**Methods:**

We conducted a multicenter qualitative study based on 30 semi-structured interviews. Participants, purposively selected until data saturation, had diverse types of cancer and had started treatment at least 6 months before interview. Data were examined by thematic analysis.

**Results:**

Our analysis found two themes: (1) what negatively affected for patient’s quality of daily life during the treatment period, a question to which patients responded by talking only about the side effects of treatment; and (2) what positively affected their quality of daily life during the treatment period with three sub-themes: (i) The interest in having —investing in — a *support object* that can be defined as an object, a relationship or an activity particularly invested by the patients which makes them feel good and makes the cancer and its treatment bearable, (ii)The subjective perception of the efficacy of the antitumor treatment and (iii) the positive effects of relationships, with friends and family, and also with their physician.

**Conclusions:**

Patients must be involved in their care if they are to be able to bear their course of treatment and find ways to endure the difficult experience of cancer care. The support object represents an important therapeutic lever that can be used by their oncologists. They should be interested in their support objects, in order to support the patients in this investment and to help them to maintain it throughout the health care pathway. Furthermore, showing interest in this topic, important to the patient, could improve the physician-patient relation without using up very much of the physician’s time.

## Background

In the field of oncology, medical advances and the development of evidence-based medicine (EBM) have produced major progress in terms of both survival and quality of care. At the same time, patients’ quality of life (QoL) has become a major objective of cancer care, considered by some authors to be the leading patient-reported outcomes for all treatment in this field [1].

There is accordingly a substantial literature on QoL and the health-related QoL of patients with cancer. It has primarily focused on:

- the impact on QoL of specific types of cancer [2–4]; or a specific treatment by surgery, radiation therapy, chemotherapy, or hormonal therapy [5–7];
- psychosocial interventions aimed at improving the QoL of cancer patients [8, 9];
- factors associated with QoL in cancer patients: anxiety and depression [10], coping strategies [11], physical exercise [12], and finances [13].

QoL in oncology is explored principally among two populations of patients: those with cancer at an advanced stage or in palliative care [14, 15] and survivors of specific types of cancers [16, 17].

Moreover, in these studies, the term Qol is understood as the results of quantitative measures to assess levels of wellness of the patient are the quality of life, that is to quantify the impact of a cancer using a psychometric approach and several concepts such as for instance physical functioning, role-physical, bodily pain, general health perceptions, vitality, social functioning, role-emotional, and mental health [18].

Exploring the quality of life of patients with cancer also requires to take an interest in their daily lives and to focus directly on their views. That requires to consider quality of life otherwise, based on the subjective experience of the patients’ day-to-day lives, what we chose to name *quality of daily life*.

Qualitative methods are useful in this context, aiming as they do to describe and understand complex phenomena in greater depth [19]. They are a tool of choice for focusing on the views of patients directly in their context. Yet, qualitative methods are rarely used among patients with cancer to address the views of cancer patients about their quality of daily life, again principally among survivors or those in advanced-stage or palliative situations [20, 21].

To our knowledge, no study has ever explored how cancer patients experience their quality of daily life during the treatment period. Their increased survival due to advances in oncology has led to longer, chronic disease and requires them to be in treatment for longer periods, with an accompanying risk of impaired quality of life on a daily basis and long-term physical and psychological effects during this period [22]. This phase of curative treatment, before the questions of survival and of palliative care arise, also affects patients’ quality of daily life.

Based on an exploration of the daily life of patients during the active treatment period, this qualitative study aimed to explore what affected their quality of daily life, either during the treatment or in their daily life.

## Methods

This exploratory national multicenter study involved oncology departments at three university hospitals, two located in the Paris area (Saint-Louis Hospital, Paris, and Avicenne Hospital, Bobigny), and one in northern France (Caen). The study design is detailed in Table 1. We used a qualitative methodology: sampling was *purposive* [23], data were collected from individual interviews, and data saturation was achieved according to the principle of theoretical sufficiency [24]. The analysis, aided by Nvivo 11 software, used a thematic approach [25], Table 2 summarizes the different stages of our thematic analysis. This study complies with the SRQR guideline [26].

**Table 1:** Design of the study

Qualitative approach	Phenomenology
Research paradigm	Constructivism
Ethical issues	The Paris-Descartes University review board (CERES) approved the research protocol (IRB number: 20140600001072). All participants provided written informed consent.
Sampling strategy	Purposive sampling strategy with maximum variation: - To include patients that differed with respect to cancer site and type, stage, duration of treatment and age. - To “challenge” the findings continuously by including participants who might invalidate what was previously found.
Recruitment strategy	- Inclusion criteria were discussed with physicians of the oncology departments where recruitment was planned. - With respect with maximum variation purposive sampling strategy, physicians were asked to identify 3 to 5 suitable patients who met the inclusion criteria - Physicians mentioned the study to potential participants and gave them an information sheet about it. - Researchers met the interestesed patients ° To describe the study ° To collect social and demographic data ° To obtain their written consent.
Participants	Inclusion criteria: - Age: 18 years or older (no upper limit) - Treatment started at least 6 months before interview - Able to communicate in French Exclusion criteria - Age: < 18 years - In the terminal phase (expected survival less than 6 months)
Data saturation	Data saturation according to the principle of theoretical sufficiency: - When new participants were not adding anything significant to the database - When the themes obtained offered a sufficient explanatory framework in view of the data collected. - Two further individual interviews were conducted with no new themes emerging, so to ensure full data saturation.
Data collection period	Between November 2014 and June 2015
Data collection methods	Individual semi-structured interviews: - To get rich and detailed personal data from each participant - Interactive conversational style - Using a list of area of experience: ° Topic 1: Story of the illness (beginning of the story) ° Topic 2: exploration of daily life during the treatment period ▪ At home ▪ In relationships ▪ At work (if maintained) ▪ In treatment - All interviews were: ° Conducted in a private room in the hospital of treatment ° Audio-recorded with participants’ permission ° Transcribed literally into verbatim. ° Anonymized
Interviewers	Experienced qualitative researchers - A psychologist (MO) - Two psychiatrists (JS, ML)
Duration of interviews	From 60 to 90 min.
Data analysis	Thematic analysis: - To identify, analyze and report themes within data (a theme = a label that summarizes the essence of a number of related codes) - To identify the similarities and the differences in the participants’ narratives. - To discern recurrent patterns and to integrate new elements that emerged from the analysis - Data-driven analysis with inductive approach = coding the data without any reference to theoretical notions or researcher’s preconceptions.
Criteria to ensure validity	Analysis conducted independently by three researchers (JS, MO, ML) - To verify that the themes identified were an exact reflection of the data. Research group monthly meetings: - To discuss the results - To be supervised by two researchers more distant from the material (ARL, LV). - After negotiation of disagreements and discrepancies within the research team during regular meetings, consensus was reached on all findings.

**Table 2:** Thematic analysis

	Activities	Rationale
Stage 1	Repeatedly read each transcript, as a whole	Obtain a global picture of the interview and become familiar with the interviewee’s verbal style and vocabulary. Each new reading of the transcript might also provide new perspectives.
Stage 2	Code the transcript by making notes corresponding to the fundamental units of meanings.	Pay particular attention to linguistic details and the vocabulary used by the participant, for instance when he/she uses a metaphor to explain or name a phenomenon, in order to make inductive descriptive notes using the participant’s own words.
Stage 3	Make conceptual notes through processes of condensation, abstraction, and comparison of the initial notes.	Categorize initial notes and reach a higher level of abstraction.
Stage 4	Identify initial themes. Provide text quotes that illustrate the main ideas of each theme.	Themes are labels that summarize the essence of a number of related conceptual notes. They are used to capture the experience of the phenomenon under study.
Stage 5	Identify recurrent themes across transcripts and produce a coherent ordered table of the themes and sub-themes.	Move from the particular to the shared across multiple experiences. Recurrent themes reflect a shared understanding of the phenomena among all participants. During this more analytic stage, researchers try to make sense of the associations between the themes found.

In the results that follow, extracts of the transcripts have been selected to exemplify the themes described. All personal information has been removed, to protect the confidentiality of the participants. The verbatim account has been freely translated into English for the sole purposes of this article.

## Results

We included 30 patients. All of the patients asked for interviews agreed to participate. Table 3 presents their social and clinical characteristics. Our sample was 57% female (M = 13, F = 17) and had a mean age of 63.5 years - from 31 to 77 years old-. The median time since diagnosis was 4 years, and all patients had undergone several different treatments.

**Table 3:** Summary of participants

	n (%)
Gender, women	17 (57)
Age, mean y	63,5
30–40	3 (10)
40–50	5 (17)
50–60	6 (20)
60–70	9 (30)
≥ 70	7 (23)
Cancer type	
Breast carcinoma	9 (30)
Lung carcinoma	1 (3)
Melanoma	7 (23)
Skin lymphoma	6 (20)
Bladder/kidney carninoma	3 (10)
Prostate carcinoma	1 (3)
Testis germ cell cancer	1 (3)
Ovaries	2 (7)
Disease stage	
Metastatic	14 (47)
Localized	16 (53)
Treatment recieved	
Intravenous chemotherapy only	6 (20)
Intravenous chemotherapy + others	19 (63)
Oral chemotherapy, other treatments	5 (17)
Duration of cancer treatment period	
Less than 1 year	6 (20)
1 to 3 years	6 (20)
3 to 5 years	12 (40)
More than 5 years	6 (20)
Recruitment site	
Paris St Louis Hospital	15 (50)
Bobigny, Avicenne Hospital	3 (10)
Caen, university hospital centre	12 (40)

The presentation of our results is structured by two themes: (1) what negatively affected for their quality of daily life during the treatment period, a question to which patients responded by talking only about the side effects of treatment; and (2) what positively affected their quality of daily life during the treatment period: use of a support object/activity during the treatment period, antitumor treatment, and relationships.

### What negatively affected for quality of daily life: Treatment side effects

#### The adverse effects of antitumor treatments

The patients included in our study found the adverse effects of antitumor treatments difficult to live with. First, they dreaded these effects, especially those due to chemotherapy.

*P3: “It's true that I was very afraid of chemotherapy. I said to myself that everyone who has chemo, they are very sick. They vomit. I told myself, I'm going to lose my hair, I'm going to lose weight, I'm going to vomit. I'm not going to eat.”*

*P4: “All that I knew about chemo was from movies in the 1980s where people were as sick as dogs all the time for weeks.”*

Then they complained about the side effects, such as nausea and vomiting or asthenia, which strongly affected their day-to-day quality of life. They also reported that they were unable to accomplish the tasks of daily living or to do any leisure activities.

*P10: “I could never go back to the work I did before, I wouldn't have the energy.”*

*P12: “Chemo is so powerful and so noxious at the same time that really, what a bitch when you see the side effects. I couldn't do my errands, couldn’t go shopping or to the movies for months.”*

Finally, they lamented the persistent sensory effects, such as the loss of a sense of taste, and those that impaired their self-image, such as the loss of hair, mentioned as often by men as by women.

*P17: “I had chemo, hard chemo. It made my hair fall out, it destroyed everything it went through. If you talk about the effects of chemo, it's the hair, and then the nails, and gastrointestinal problems.”*

#### The paradoxical experience of adverse effects

We also found a paradoxical experience around these side effects. Simultaneously, chemotherapy made them feel bad and had numerous burdensome effects, but these also became an indicator of its therapeutic efficacy.

*P20: “It destroys the diseased cells, with an impact that we call side effects, collateral damage.”*

*P29: “What matters is the result. We know that chemo kills good cells, but it also kills the bad ones.”*

Similarly, we observed patients’ ambivalence toward invasive treatment such as surgery, experienced as burdensome and dangerous but also as radical and more effective.

P23: “*I had a rotten bladder, they took it out, but the party goes on, if I can say it like that*.”

P28: *“The lung operation was hard to stand, you don't recover just like that, but then, afterwards, it went better.”*

### What positively affects the quality of daily life

Patients’ narratives about what allowed them to maintain a good quality of daily life on a daily basis during treatment were richer and more varied. They emphasized three different aspects:

- (i) The interest in having —investing in — a *support object*. We have chosen to translate the French idiom “objet d’étayage” by the English term *Support object*. *“Objet d’étayage”/support object* can be defined as an object, a relationship or an activity particularly invested by the patients in their daily lives, which makes them feel good and makes the cancer and its treatment bearable.
- (ii) Their subjective perception of the efficacy of the antitumor treatment also exerted a positive impact on their quality of daily life.
- (iii) The positive effects of relationships.

#### The support object

Most of the patients had an activity or relationship especially important to them that was good for them and helped them to live better with their disease and its treatment. This real function of this *object* was to support them.

*P1: “My camping car. That's what saved me. I love to travel! Roaming, I love it.”*

For some patients, this was a regular physical activity. They described a physical effect, that is, an awareness of their physical capacity. They also mentioned a moment of escape, where they were not thinking about their disease and, for those who did team sports, the ability to maintain a social association outside of a health context.

*P27: “I began to play golf too: to let go and not think about anything. And that, it was really good. It's always really good, it lets you think only about yourself, already, a little, and to escape from all that. It lets you really concentrate, to empty your head and not think about anything but yourself.”*

For other patients, it was a hobby or traveling, again something that let them take themselves out of the everyday and escape from the disease and from treatment.

*P10: “What helped me most was all the beautiful performances I saw, which are still like a small fire inside me.”*

For others, it could be work, or religious or spiritual practice, or meditation.

*P7: “I'm a deputy mayor in my municipality; [the cancer] doesn't keep me from going to spend two hours a day at city hall and participating in town council meetings. I live normally.”*

*P30: “Yoga with its Hindu philosophy, it really puts things in perspective.”*

#### Subjective perception of the effectiveness of antitumor treatment

What positively affected patients’ quality of daily life, according to them, was perceiving that their antitumor treatment was effective. They had a representation of cancer treatment as a battle against the disease; treatment was perceived as effective if it halted the disease and ineffective if it did not.

*P11: “Chemo, that helped me. Stopped the bad stuff.”*

*P7: “I cannot say that the treatment is helpful today, because I have more lesions today than I did two months ago.”*

Some patients relied on and appropriated clinical or paraclinical efficacy criteria, such as tumor size on imaging or the lab measurements of tumor markers.

*P2: “When the PSA goes down, I'm in a better mood, it makes me happy.”*

The type of treatment also influenced the perception of efficacy. Patients found it easier to perceive surgical treatment as effective. Patients described surgery as a one-time procedure with generally a curative objective: total ablation of the tumor.

*P18: “As soon as they told us the day after the operation that it had succeeded, all we had to do was wait to get better. Finally it wasn't so awful.”*

Inversely, patients perceived the unavailability of surgical treatment as inefficacy.

*P5: “But they didn't operate on me. Because otherwise, I said, take off both breasts and we're done. But apparently it wasn't possible, it was inoperable.”*

#### Relationships

Relationships had an important place in patients’ discourse.

The presence of family and close friends was very important for the patients, so that they were not alone and so that they had support during treatment, and also in their daily life.

*P3: “What's sure is I had my friend. He came each time I had chemo. He was with me. It's true that, if I had been all alone, at home, it would have been harder. He helped me.”*

The relationship with the physician was also very important. What was helpful, according to the patients, was the establishment of a trusting relationship with the physician together with the latter’s involvement and availability.

*P10: “I know that he is someone who is very committed, very involved, who won't let me down.”*

Patients were also very sensitive to the physician’s ability to listen and to reassure.

*P17: “And it's also the difference between a good and a bad doctor, because behind that it means he’s listening.”*

The quality of the relationship appeared more important than the time spent with the patient or the information provided.

*P18: “I never need to spend very long with a doctor, finally. I often go right to the essential part. We all know that we're not the only person they are taking care of, but, no, I never had the impression I'm being rushed.”*

The patients also reported positive effects of a convivial and sympathetic treatment environment, directly associated with their relationship with the health care team and especially the nurses.

*P17: “Support from the nurses has always been great. I don't know how they do it, but they manage to say something nice to everyone. To show their interest, and even to remember people.”*

Finally, relationships with the other patients were also experienced as helpful, when it provided hope, mutual assistance, and friendliness.

*P4: “She told me she had had a remission for 18 years. But suddenly, there was hope. If she could have 18 years, I can too.”*

*P18: “Twice I had a roommate who I got along well with, who I talked to. Yes that helps pass the time, it's always nice.”*

## Discussion

Our results are structured around the dialectic with, on the one hand, what negatively affected quality of daily life, which for all patients was always only the side effects of treatment, and on the other hand, what had a positive effect on quality of daily life, including a support object or activity, the perceived efficacy of the treatment, and relationships.

The only topic covered in the patient’s narratives of things that negatively affected their quality of daily life was side effects, which recurred over and over, invading their discourse throughout the interviews. Both the ubiquity and the invasiveness of side effects in the patients’ experience lead us to discuss two specific points: the potentially post-traumatic dimension of this experience of side effects and the importance of daily life for the patients.

Several studies have found a relatively substantial frequency of post-traumatic symptoms, and even of full post-traumatic stress disorder (PTSD) in patients with cancer [27]. PTSD is a mental health condition triggered by a traumatic event, either experienced or witnessed. Symptoms include intrusive memories (flashbacks) and uncontrollable thoughts about the event, nightmares, avoidance reactions, severe anxiety, negative mood and emotional reactions. PTSD cause significant social distress and can lead to other psychiatric issues such as depression [28]. A meta-analysis found a lifetime prevalence of cancer-related PTSD of 12.6% [29]. The adverse effects of antitumor treatments are reported to be a potentially determinant factor of these symptoms but are not considered as a separate experience in the patient’s history. Our results suggest, nonetheless, that the experience of the side effects of antitumor treatment in these patients could have a specific post-traumatic dimension, and could be considered as a traumatic stressor [30], that may thus require targeted screening, evaluation, and interventions.

There is a substantial literature describing the influence of these side effects on treatment decisions [31] as well as, especially, their negative impact on patients’ QoL [32–34]. Prolonging treatment and focusing only on survival, does not meet all patients’ needs [35]. In our results, the patients underlined the importance of living with cancer on a day-to-day basis, that is, not only living as long as possible, but also as well as possible in their everyday life.

The importance of interpersonal relationships and of the quality of the physician-patient relationship has been known and reported repeatedly in the literature [36, 37]. Our results describe an original aspect of what directly and positively affects patients’ quality of daily life: involvement with a support object. The support object, in our opinion, represents an important therapeutic lever that can be used to improve the physician-patient relationship. That is, when patients are able to choose and be involved with a support object or activity, the physician must support them and converse with them on this topic.

### Clinical implications

Numerous studies have showed that physicians fail to take the spiritual and religious concerns of their severely ill patients into account [38], despite the demonstrated benefits in terms of patients’ satisfaction and the importance of these aspects to their QoL [39]. In the field of cancer care, physicians should be interested in their patients’ support objects, whatever they are. First, this allows doctors to support the patients in their investment in this object and to help them maintain this investment throughout the health care pathway. Second, showing interest during visits in this topic, important to the patient, and conversing about it could help to establish a trusting relationship and therefore, according to our results, improving his or her quality of daily life, without using up very much of the physician’s time. That is, with the increasing transformation of cancer into a chronic disease comes the need for a different kind of relationship, one in which physicians can fulfill the relational needs of care. The issue here is not that oncologists should replace psychologists or psychiatrists [40], but rather that they have a relational tool that enables them to support the patient in their management of a serious and very trying chronic disease and thus to help them to maintain their quality of daily life. We chose to focus on patients’ daily life during their treatment period. This differs from, and is complementary with, others approaches such as shared decision making [41] or self-management [38], that also seek to improve patients’ quality of daily life and patient-physician relations.

### Study limitations

Our qualitative study took place in three different centers and included patients with various types of cancer. These points make our findings transferable to other cancer contexts. However, some limitations must be taken into consideration. First, this took place in France, and caution is required in transposing our results to other places, especially non-Western countries, because cancer care depends strongly on the organization of the medical system as well as on the country’s economy. Second, our recruitment process did not allow us to include patients who have dropped out of treatment or those using complementary or alternative treatment, although they are relatively frequent in this clinical population [42]. This might have limited our findings. Third, all patients had undergone several treatments for at least 6 months and the median time since diagnosis in our sample was 4 years. Our results might not be relevant for patients who have just started treatment. Further qualitative studies should be made to address this specific period of time. Finally, our results don’t mention the influence of age on the quality of daily life. Further qualitative studies with specific age populations should be made to explore the extensive role age plays in this matter.

## Conclusion

Quality of life is a daily concern for patients during cancer treatment. This qualitative study provides access to patients’ experience of their care and daily lives, allowing us to see cancer care through the patients’ eyes. In their daily clinical practice, doctors face multiple constraints – of time and workload —that hinder them from taking their patients’ subjective health status into account. We suggest as a start that they include in their practice a systematic interest for the support object that their patients have chosen.

## Abbreviations

EBM: Evidence Based Medicine
PTSD: Post-traumatic stress disorder
QoL: Quality of life
